# The people-centered care and inpatients’ perceived experience in China: a nationwide cross-sectional study

**DOI:** 10.1186/s12939-025-02409-2

**Published:** 2025-02-18

**Authors:** Zhixing Wang, Xueyao Wang, Herng-Chia Chiu, Xiangrong Kong, Qingfeng Li, Xu Ran, Yang Liu, Hailun Liang, Leiyu Shi

**Affiliations:** 1 Department of Health Policy and Management, Johns Hopkins Bloomberg School of Public Health, Baltimore, 21205 USA; 2 Institute of Social Security, School of Public Administration and Policy, Renmin University of China, Beijing, 100872 China; 3 Institute of Hospital Management, Tsinghua University, Beijing, 100084 China; 4 Department of Epidemiology, Johns Hopkins Bloomberg School of Public Health, Baltimore, 21205 USA; 5 Department of International Health, Johns Hopkins Bloomberg School of Public Health, Baltimore, 21205 USA; 6 Doctor-Patient Experience Research Base, National Health Commission of the People’s Republic of China, Beijing, 100044 China; 7 Department of Public Health, School of Population and Health, Renmin University of China, Beijing, 100872 China

## Abstract

**Background:**

The concept of People-Centered Care (PCC) is a prominent concept around the world, which is considered as an important concept and practice to promote health equity especially in China. Nevertheless, the association between PCC and the perceived experience of patients remains unclear, particularly from the perspective of the entire nation. This study examined the relationship between PCC and inpatients’ perceived experience in China.

**Methods:**

The study utilized nationwide data collected from 351 healthcare facilities in 31 provinces representing all facility levels and types using proportional odds models. The five attributes of PCC encompass the following categories: continuity of care, information sharing, enhanced access, effectiveness, and respect, each contributing to improving health equity. Inpatients’ perceived experience includes the following factors: inpatients’ satisfaction with the hospitalization, the recognition of the hospital, and the recommendation of the hospital.

**Results:**

Concerning inpatients’ overall satisfaction with the hospitalization, all PCC attributes had a positive effect on satisfaction, especially for inpatients with higher levels of care continuity and respect, contributing to health equity. Inpatients with a higher level of continuity were 3.66 times more likely to ameliorate their level of satisfaction from “very unsatisfied” to “unsatisfied.” Meanwhile, all PCC attributes had significantly positive effects on inpatients’ recognition, with effectiveness and respect showing an even stronger association with health equity. Regarding inpatients’ recommendation measures, all PCC attributes were positively associated, especially with higher levels of care continuity and effectiveness.

**Conclusion:**

People-centered care is positively associated with inpatients’ perceived experience, and enhancing health equity through PCC attributes can further improve this experience. Further reform and practice should focus on the amelioration of continuity of care, promotion of information sharing between medical staff and patients, access and effectiveness of care, and respect for patients, all contributing to health equity.

## Background

With the transformation of the traditional disease-centered medical model, the emergence of People-Centered Care (PCC) puts emphasis on the feelings, values, and participation of patients, their families, and communities in order to ensure a reasonable and effective supply of services. Enhancing patient participation in service provision is of extraordinary significance and is valuable concerning the improvement of efficiency, effectiveness, accessibility, and fairness of medical services, particularly in an aging society that is seeing a shift in the disease spectrum towards chronic diseases. PCC, as an essential concept in the current healthcare system, has been recognized and accepted by countries around the world [1]. Numerous countries have also developed assorted PCC models based on their individual differences. According to the World Health Organization (WHO), people-centered care is "an approach to care that scientifically accommodates the perspectives of individuals, families, and communities and sees them as participants as well as benefits of trusted health systems that respond to their needs and preferences in human and psychological ways" [2]. Various PCC models have also been promoted in the United States. For example, the patient-centered medical home was a practice that included a foundation built on PCC which aimed to provide patient-centered, comprehensive, coordinated, accessible health care and committed to quality and safety [3]. The National Committee for Quality Assurance (NCQA) also addresses the attributes of PCC including patient-centered access and continuity, care coordination, care transitions, and performance measurement and quality improvement [4]. PCC emphasizes designing and providing healthcare services around patients' preferences, values, needs, and expectations, fully considering patients' living conditions, psychological state, family support, and their understanding of the disease, in order to better ensure that the treatment plan is accepted and followed by the patient. PCC focuses on the effectiveness of the interaction between healthcare providers and patients during the service delivery process, ultimately improving the quality and effectiveness of healthcare services.

In China, PCC is understood from five aspects, including the continuity of health services, the promotion of information sharing, enhanced access, effectiveness, and respect. This understanding aligns with China's policies and practices. China has issued a large number of policies and measures to promote the patients’ experience through the promotion of PCC [5]. Since the new round of health reform in China in 2009, the government has put forward a series of strategies aimed at improving the patient experience in all levels of hospitals including tertiary hospitals, secondary hospitals, and community healthcare organizations, and PCC has gradually found a place as one of the core concepts that guide China’s health system reform [6, 7]. In 2016, The World Bank, WHO, and the Chinese government jointly published a report that emphasized the value of PCC and promoted a model of People-Centered Integrated care, which resulted in the creation of integrated care policies and public hospital reform policies [8, 9]. These two series of policies focused on PCC entirely and had the goal of promoting PCC utilizing five factors:

The first of which was to improve the continuity of health services through the collaboration of various levels of health organizations [10]. At the county level, rules related to referrals have been implemented, and the responsibilities of health organizations at all levels have been given clarification [11]. This provides transparent behavioral norms and a clear basis for health organizations and medical staff to guarantee timely and accurate referrals [12]. Second, the promotion of information sharing between the patients and the medical staff and empowerment of the patients to engage in the decision-making and health services coproduction has been emphasized which in turn makes this healthcare process easier to transition through during an appointment with medical staff or in the case of hospitalization [13]. Third, creating a situation where health services are increasingly accessible through health networks and resource sharing between various health organizations has been addressed. According to national policies, each county should launch a healthcare network through a hierarchical system or contracting. Through a strengthening of the assistance given to tertiary and secondary hospitals and in turn to community health organizations, the reform aims at enhancing the service capacity and level of all hospitals in a county. Fourth, an improvement in the effectiveness of services is set as a priority. By investigating the level of patient satisfaction and their self-assessment of health, the results of the investigations are able to provide a foundation for the reform of hospital management, service supply, and doctor attitudes, and ultimately an improvement to the effectiveness of services. Fifth, the final aspect focuses on making the patients feel a higher level of respect [14].

A lot of researches has stressed the impact of PCC. Existing research suggests that PCC can improve patient satisfaction. PCC enhances the overall satisfaction of patients by improving the doctor-patient relationship and providing personalized treatment plans. In hospitals implementing PCC, patients tend to rate the environment, nursing quality, and the attitude of healthcare professionals more highly [15]. Additionally, PCC can enhance patients' emotional experiences and mental health, alleviating anxiety, fear, and loneliness during hospitalization [16]. PCC is also believed to improve patient adherence to treatment, as patients with higher evaluations of PCC are more likely to follow medical advice actively. In other words, PCC can improve patients' evaluations and health outcomes across various dimensions [5, 17]. Furthermore, research has also explored the relationship between PCC and health equity [18]. Studies have shown that PCC promotes health equity through personalized and inclusive care services, encouraging patient involvement in decision-making, respecting cultural differences, addressing vulnerable groups and social determinants, improving accessibility to healthcare services, and emphasizing prevention and health promotion [19–21]. These measures help eliminate inequalities and injustices in healthcare services, ensuring that everyone can access the necessary medical support and services equally, particularly for socially disadvantaged groups. The PCC model plays a significant role in promoting health equity.

Meanwhile, the association between PCC and patients’ perceived experience has drawn tremendous scholarly attention. According to contemporary research, PCC significantly impacts patients' perceived experience. A significant amount of research has explored the association between PCC and patients’ perceived experience, however, the specific effects remain unclear. According to research conducted in 56 primary care sites of the Veterans Health Administration, PCC was not associated with the patient experience of care [22]. Other studies of PCC practices supported this conclusion as well [23–26]. In additional studies, higher PCC scores and higher levels of PCC had a significantly positive effect concerning patient experience including patients from primary care organizations and hospitals [27–32]. Nonetheless, a study of pediatric and family medicine PCMH discovered that the adoption of PCMH which aimed at improving the level of PCC may in fact worsen the experience of patients [33]. The contemporary research presented that the effect of patient experience was one of heterogeneity which the effect of PCC on patient experience remained unclear. This implies necessitates further research so as to be addressed properly.

Contemporary research has shown that there exists a correlation between PCC and the patients’ perceived experience. Even so, the impact of PCC on the patients’ perceived experience in the context of the Chinese healthcare system is not clear, which may hinder the further reform of the Chinese healthcare system and the improvement of the patients’ perceived experience. When taking into consideration the gap that exists in contemporary research, this study aims to investigate the association between PCC and the patients’ perceived experience using the data from the China Patient Self-Reporting Experience Survey (CPSRES) in 2023 in order to address this study’s research question [34]. The survey currently covers 31 provinces, autonomous regions, and municipalities directly under the administration of the central government of China, which is the first standardized, large-scale, and continuous survey of patients’ perceived experience nationwide utilizing the support of big data, which can, in turn, provide a concrete foundation to address the pertinent research question.

## Methods

### Data source

The Patient Self-Reporting Experience Survey is a nationwide survey aimed at surveying patients from hospitals in assorted provinces across the country and then evaluating their experiences. The survey was carried out under the supervision of the National Health Commission and aimed to monitor the patient experience among patients within the Chinese healthcare system at all levels of health organizations. The stated goal was to assist hospitals in improving the quality of healthcare services. The questionnaire of the survey was tested to have excellent reliability and validity [35]. This survey was carried out in more than 1000 health organizations in total from 31 provinces, municipalities, and autonomous regions beginning in 2019. Thus far, more than 17.74 million individuals have participated in this survey. The participants of the survey included both outpatients and inpatients who received medical treatment in hospitals and a totality of the participants of the survey were over the age of 18 years [36]. All the participants of the survey were capable of completing all the questions on a mobile server individually on their own or in conjunction with the help of an investigator. If a situation arose where patients were unable to complete the questions individually because of severe mental disorders, coma, dementia, or other debilitating disorders, then family members were able to answer the questions on the patient’s behalf.

### Study sample

The data used in the study included the patients’ perceived experience in 2023. The sampling strategy of this study is primarily divided into two steps: the first step is the division of all cities into three levels of high, medium, and low levels of per capita GDP based on the per capita GDP of all prefecture-level cities in each province in 2022. Two cities are selected from each assorted level according to the principle of random sampling, and an entirety of the hospitals in those sampled cities are included. The second step is to draw 300 patients from each hospital. Sample patients were found in the hospital's sample pool. If the number of patients surveyed by the hospital was less than 300, all patients were included. If the number of patients surveyed by the hospital was higher than 300, stratified sampling was conducted by age. Each institution then selected 24 patients under the age of 18, 56 patients aged 19–39, 99 patients aged 40–59, 99 patients aged 60–79, and 22 patients aged 80 and above. Some of the samples included in this study were from proxy-reported patients. The final sample includes 194 tertiary hospitals, 141 secondary hospitals, 16 community health organizations, and 84,438 inpatients.

### Measures

#### Outcome measures

The outcome of this study is the inpatients’ perceived experience, which includes three indicators: the inpatients’ overall satisfaction with the hospitalization, the inpatients’ recognition of the hospital, and the recommendation of the hospital. The overall satisfaction of the hospitalization is measured by the question “Were you satisfied with your overall experience of this hospitalization?” Additionally, the answers included “very dissatisfied”, “dissatisfied”, “average”, “satisfied”, and “very satisfied”. In order to measure the inpatients’ recognition, the respondents were asked “Would you choose this hospital again if you needed healthcare services?” The answers included “definitely won't”, “won't”, “average”, “will”, and “definitely will”. The measurement of the recommendation is founded on the question “Would you recommend this hospital to your friends and family when they need healthcare services?,” and the answers also included “definitely won't”, “won't”, “average”, “will”, and “definitely will”.

#### People-centered care

The independent variable of the study is people-centered care (PCC). Based on prior research and the questionnaire of the survey, the PCC of the study encompassed five attributes, which are continuity, information sharing, enhanced access, effectiveness, and respect. The entirety of these attributes was measured utilizing questions with answers ranked 1,2,3,4 and 5.

The reason that this study chooses to measure PCC utilizing the five attributes is that China’s policy and relevant strategies primarily focused on the above domains in the promotion of PCC in China. As far as continuity, China launched an integrated healthcare system policy in 2017 as a national policy to promote the continuity of health services through horizontal and vertical integration of health organizations with the goal of eventually improving the patients’ experience which is one of the primary stated goals of the reform. Furthermore, China vigorously promotes the construction of information systems to guarantee that the information on health services including examination results, as well as cost information, can be delivered to patients in a timely manner, which in turn reduces the information asymmetry between doctors and patients. Advanced information systems also facilitate doctors to timely inform patients, consequently promoting information sharing between doctors and patients. As to improved access, documents including the High-Quality Development of Public Hospitals regard improving hospital service quality as a crucial strategy in promoting patient experience and satisfaction. In the preliminary research and data analysis, it was discovered that in China, the principal reason for patients to choose a hospital for treatment is the high quality of health services provided at that hospital.

Consequently, patients' perception of health service quality is considered an essential part of PCC in China. Effectiveness reflects the patients’ self-evaluation of the health outcomes of health services received, thus it is a crucial attribute of the PCC in China. Simultaneously, the reform of China’s hospitals focuses on the investigations of patient satisfaction, and respect is an important aspect of patient satisfaction investigations. Relevant policies in China prioritize the promotion of respect as a pivotal element in the improvement of patients’ satisfaction and reducing conflicts between doctors and patients. Consequently, the study includes respect as the fifth attribute of PCC. Therefore, combining China's practical experience, the current focus of reforms, and policy objectives, we use continuity, information sharing, enhanced access, effectiveness, and respect to measure PCC. This is primarily because the data collection and research are conducted within the context of China's health reform. Therefore, the measurement of PCC must consider the characteristics and policy goals of the current reforms in China. Measuring PCC across these five dimensions enhances our understanding of China's related reforms, enabling the research findings to further provide insights for China's ongoing reforms.

The attributes and corresponding items are displayed in Table 1.

**Table 1:** People centered care attribute and items

PCC attributes	Items
Continuity	Do you think the admission procedure was smooth?
	Are you satisfied with the overall service flow of hospitalization?
	Are you satisfied with the internal signage and the clarity of instructions?
Information sharing	Are you satisfied with the doctor's information about your diagnosis and 1condition?
	Are you satisfied with the doctor's information about the treatment plan, prognosis and medical risks?
	Did the medical staff introduce to you the use of medication, adverse effects and precautions clearly?
Enhanced access	On the day you were admitted to the hospital, were you satisfied with the meticulousness of the doctor's first examination?
	How satisfied were you with the doctor's attention to detail during the room visit?
	What do you think of the general level of treatment by the doctor?
Effectiveness	When you experience pain or discomfort, are you satisfied with the care and treatment provided by the medical staff?
	Are you satisfied with the improvement of your symptoms through treatment?
Respect	Are you satisfied with the protection of your privacy by the medical staff during examination or treatment?
	Are you satisfied with the doctor's service attitude?
	Are you satisfied with the nurses' service attitude?

To test the internal consistency of the attributes of PCC, composite measures were calculated and the Cronbach’s α scores were as follows: continuity(0.80), information sharing(0.83), enhanced access(0.88)m effectiveness(0.82), and respect(0.87). In this study, the Variance Inflation Factor (VIF) was employed to test the multicollinearity among independent variables. Through calculation, it was found that the VIF values of Continuity, Information sharing, Enhanced access, Effectiveness, and Respect were 3.51, 3.24, 3.05, 3.19, and 3.58 respectively. All these values are less than 10, indicating that there is no multicollinearity among the five dimensions of PCC.

*Continuity* is measured by three questions: (1) Do you think the admission procedure was smooth? (very smooth, smooth, fair, not smooth, very unclear), (2) Are you satisfied with the overall service flow of hospitalization? (Very Satisfied, Satisfied, Average, Dissatisfied, Very Dissatisfied), (3) Are you satisfied with the internal signage and the clarity of instructions? (Very Satisfied, Satisfied, Average, Dissatisfied, Very Dissatisfied).

*Information sharing* is also measured by three questions: (1) Are you satisfied with the doctor's information about your diagnosis and condition? (Very Satisfied, Satisfied, Average, Dissatisfied, Very Dissatisfied), (2) Are you satisfied with the doctor's information about the treatment plan, prognosis, and medical risks? (Very Satisfied, Satisfied, Average, Dissatisfied, Very Dissatisfied), (3) Did the medical staff introduce to you the use of medication, adverse effects, and precautions clearly? (Introduced, very clear, Introduced, basically clear, Introduced, not very clear, Introduced, not clear, Not introduced).

*Enhanced access’s* measurement consists of three questions: (1) On the day you were admitted to the hospital, were you satisfied with the meticulousness of the doctor's first examination? (Very Satisfied, Satisfied, Average, Dissatisfied, Very Dissatisfied) (2) How satisfied were you with the doctor's attention to detail during the room visit? (Very Satisfied, Satisfied, Average, Dissatisfied, Very Dissatisfied) (3) What do you think of the general level of treatment by the doctor? (very good, good, average, bad, very bad).

*Effectiveness* was measured by: (1) When you experience pain or discomfort, are you satisfied with the care and treatment provided by the medical staff? (Very Satisfied, Satisfied, Average, Dissatisfied, Very Dissatisfied) (2) Are you satisfied with the improvement of your symptoms through treatment? (Very Satisfied, Satisfied, Average, Dissatisfied, Very Dissatisfied).

*Respect* is measured by the following three questions: (1) Are you satisfied with the protection of your privacy by the medical staff during examination or treatment? (Very Satisfied, Satisfied, Average, Dissatisfied, Very Dissatisfied) (2) Are you satisfied with the doctor's service attitude? (Very Satisfied, Satisfied, Average, Dissatisfied, Very Dissatisfied) (3) Are you satisfied with the nurses' service attitude? (Very Satisfied, Satisfied, Average, Dissatisfied, Very Dissatisfied).

Referring to the existing research [37], every participant’s scores on all the questions of each attribute were summed, and the mean of the total scores of each attribute was calculated. Based on the means, continuity, information sharing, enhanced access, effectiveness, and respect were processed as a dichotomous variable. The participants with a score of or greater than the mean score were coded as 1 (a higher perception of that PCC attribute) and those below were coded as a 0 (a lower perception of the PCC attribute).

#### Covariates

The people-centered care (PCC), along with the effect of PCC on the patients’ experience has drawn the attention of numerous researchers recently. In the relevant body of research, it was found that sociodemographic characteristics including age, sex, education status, long-term residence, patient cost categories, occupation, and annual household income were associated with the patients’ experience. In addition to sociodemographic characteristics, institutional characteristics may also be an important factor because they may affect the patient’s experience. Referring to the contemporary research and the questionnaire of the survey, eight sociodemographic characteristics and three institutional characteristics were included as covariates and can be seen as follows.

##### Sociodemographic characteristics

Eight sociodemographic characteristics were included: sex (female or male), age (80 years and above, 60–79 years, 40–59 years, 19–39 years, or under 19 years), the residence (local city, other cities in the province, other provinces, Hong Kong, Macao, Taiwan, and overseas), insurance (urban employee medical insurance, medical insurance for urban and rural residents, public funded health care, commercial insurance, out of pocket, and others), occupation (students; company employees, corporate executives, workers, farmers, civil servants, military personnel, individual operation, unemployed, retired, self-employed, and others), Annual household income (under 30,000 yuan, 30,000–100,000 yuan, 100,000–200,000 yuan, 200,000–500,000 yuan, and 500,000 yuan and above), the most essential reason for choosing the hospital (the hospital is well-known, utilizes high technology, and advanced equipment, a positive service attitude, a positive environment, nearby, a reasonable level of fees, introduced by other people or there are acquaintances in the hospital or others), referral or not (referral from higher level hospitals, referral from hospitals of the same level, referral from lower level hospitals, referral from community clinics, and directly coming to the hospital).

##### Institutional characteristics

 The hospital system in China exhibits a hierarchical structure, with significant differences in the medical resources and service capabilities available at different levels of hospitals. Therefore, the levels of hospitals is an important influencing factor, including tertiary hospitals, secondary hospitals and community health centers. In addition, institutional characteristics also include types of the hospitals (gynecology hospitals, traditional Chinese medicine hospitals, general hospitals, tumor hospitals, others) and hospital ownership (private, public).

### Statistical analysis

Statistical analyses were conducted by utilizing Stata version 16.0 (StataCorp LLC. Texas, USA). Sociodemographic information, PCC, and the patients’ experience were shown through the utilization of descriptive statistical analysis, including both frequency and proportion. The association between PCC and the patients’ experience was estimated by performing proportional odds models and utilizing the odds ratio (OR) and 95% confidence intervals (CIs).

## Results

### The sociodemographic and institutional characteristics

Tables 2, 3, and 4 characterizes the sociodemographic and institutional characteristics of the 84,438 sampled inpatients participating in the survey. Of all the participants, 48.72% were female, and 33.69% of the sampled inpatients were in the age group of 40–59, followed by the age group of 60–79 (31.82%). The majority of the sampled inpatients came from the local residents (86.30%). Of the total number of participants, 45.90% had Urban Employee Medical Insurance. When considering occupation, 33.80% of the sampled inpatients were either retired or freelancers, 21.68% were students, and 20.05% were farmers. 57.97% of the participants’ income per year was under 30,000 yuan RMB, and 32.41% of the participants’ income per year was between 30,000 and 100,000 yuan RMB. The results present that the primary reason to be considered when selecting the current healthcare institution is the service quality (24.70%), and the second reason is the geographical accessibility of the institution. 75.49% of the participants directly arrived at the institution without a referral. 63.28% of the participants were from tertiary hospitals, 35.06% of the participants were from tertiary hospitals, and 1.66% were from community health organizations. Considering the types of institutions, 91.19% of the participants came from general hospitals, and 93.57% of the participants were sampled from public hospitals.

**Table 2:** The sociodemographic and institutional characteristics of the sampling inpatients’ satisfaction in China

	Satisfaction(%)				
	Very dissatisfied	Dissatisfied	Average	Satisfied	Very satisfied
**Sex**					
Male	38(0.05)	153(0.18)	2,073(2.46)	22,213(26.31)	16,660(19.73)
Female	48(0.06)	147(0.17)	2,233(2.64)	23,506(27.84)	17,367(20.57)
**Age**					
Under 18	12(0.01)	22(0.03)	387(0.46)	3,218(3.81)	2,712(3.21)
19–39	15(0.02)	65(0.08)	1,028(1.22)	8,677(10.28)	7,142(8.46)
40–59	16(0.02)	98(0.12)	1,407(1.67)	15,744(1.67)	11,186(13.25)
Above 60	43(0.05)	115(0.14)	1,484(1.76)	18,080(21.41)	12,987(15.38)
**Residence**					
Local city	72(0.09)	245(0.29)	3,708(4.39)	40,349(47.79)	28,495(33.75)
Other cities in the province	1(0.00)	18(0.02)	225(0.27)	2,531(3.00)	2,620(3.10)
Other provinces	10(0.01)	37(0.04)	361(0.43)	2,793(3.31)	2,868(3.40)
Hong Kong, Macao Taiwan	0(0.00)	0(0.00)	9(0.01)	31(0.04)	23(0.03)
Overseas	3(0.00)	0(0.00)	3(0.00)	15(0.02)	21(0.02)
**Insurance**					
Urban employee medical insurance	55(0.07)	167(0.20)	2,357(2.79)	21,462(25.42)	14,722(17.44)
Medical insurance for urban and rural residents	26(0.03)	96(0.11)	1,464(1.73)	20,489(24.27)	15,393(18.23)
Public funded health care	0(0.00)	2(0.00)	40(0.05)	185(0.22)	192(0.23)
Commercial insurance	0(0.00)	0(0.00)	24(0.03)	138(0.16)	160(0.19)
Out of pocket	3(0.00)	12(0.01)	211(0.25)	1,574(1.86)	1,174(1.39)
Others	2(0.00)	23(0.03)	210(0.25)	1,871(2.22)	2,386(2.83)
**Occupation**					
Students	47(0.06)	115(0.14)	1,705(2.02)	11,571(13.70)	4,868(5.77)
Company employees	2(0.00)	21(0.02)	228(0.27)	2,907(3.44)	3,270(3.87)
Corporate executives	2(0.00)	2(0.00)	29(0.03)	333(0.39)	354(0.42)
Workers	1(0.00)	16(0.02)	217(0.26)	2,698(3.20)	2,719(3.22)
Farmers	14(0.02)	50(0.06)	678(0.80)	9,486(11.23)	6,701(7.94)
Civil servants	0(0.00)	5(0.01)	36(0.04)	375(0.44)	648(0.77)
Military personnel	0(0.00)	1(0.00)	17(0.02)	129(0.15)	360(0.43)
Individual operation	4(0.00)	3(0.00)	59(0.07)	747(0.88)	807(0.96)
Unemployed	3(0.00)	11(0.01)	227(0.27)	2,683(3.18)	1,751(2.07)
Retired, self-employed, others	13(0.02)	76(0.09)	1,110(1.31)	14,790(17.52)	12,549(14.86)
**Income**					
Under 30,000 yuan	67(0.08)	189(0.22)	3,025(3.58)	28,264(33.47)	17,400(20.61)
30,000 −100,000 yuan	16(0.02)	92(0.11)	989(1.17)	13,807(16.35)	12,461(14.76)
100,000–200,000 yuan	2(0.00)	13(0.02)	228(0.27)	3,042(3.60)	3,122(3.70)
200,000–500,000 yuan	1(0.00)	5(0.01)	47(0.06)	454(0.54)	805(0.95)
500,000 yuan and above	0(0.00)	1(0.00)	17(0.02)	152(0.18)	239(0.28)
**Reason**					
The hospital is well-known	4(0.00)	29(0.03)	494(0.59)	7,130(8.44)	8,268(9.79)
The high technology, and advanced equipment	10(0.01)	66(0.08)	794(0.94)	10,840(12.84)	9,148(10.83)
Good attitude	13(0.02)	21(0.02)	378(0.45)	5,062(5.99)	5,782(6.85)
Good environment	4(0.00)	22(0.03)	294(0.35)	2,624(3.11)	1,335(1.58)
Nearby	16(0.02)	80(0.09)	1,193(1.41)	12,838(15.20)	6,583(7.80)
Reasonable fees	1(0.00)	5(0.01)	189(0.22)	1,575(1.87)	537(0.64)
Introduced by other people, there are acquaintances in the hospital, others	38(0.05)	77(0.09)	964(1.14)	5,650(6.69)	2,374(2.81)
**Referral**					
Referral from higher level hospitals	47(0.06)	112(0.13)	1,595(1.89)	10,522(12.46)	3,778(4.47)
Referral from hospitals of the same level	3(0.00)	9(0.01)	90(0.11)	766(0.91)	911(1.08)
Referral from lower level hospitals	1(0.00)	10(0.01)	113(0.13)	1,087(1.29)	1,218(1.44)
Referral from community clinics	0(0.00)	5(0.01)	33(0.04)	212(0.25)	184(0.22)
Directly come to the hospital	35(0.04)	164(0.19)	2,475(2.93)	33,132(39.24)	27,936(33.08)
**Level**					
Tertiary hospital	0(0.00)	11(0.01)	173(0.20)	1,023(1.21)	197(0.23)
Secondary hospital	48(0.06)	104(0.12)	1,811(2.14)	18,612(22.04)	9,031(10.70)
Community health centers	38(0.05)	185(0.22)	2,322(2.75)	26,084(30.89)	24,799(29.37)
**Type**					
Gynecology hospital	2(0.00)	10(0.01)	168(0.20)	1615(1.91)	959(1.14)
Traditional Chinese medicine hospital	3(0.00)	3(0.00)	121(0.14)	1,459(1.73)	909(1.08)
General hospitals	71(0.08)	275(0.33)	3,907(4.63)	41,384(49.01)	31,351(37.13)
Tumor hospital	0(0.00)	2(0.00)	61(0.07)	1,019(1.21)	693(0.82)
Other	10(0.01)	10(0.01)	49(0.06)	242(0.29)	115(0.14)
**Ownership**					
Private	3(0.00)	16(0.02)	357(0.42)	2,845(3.37)	2,210(2.62)
Public	83(0.10)	284(0.34)	3,949(4.68)	42,874(50.78)	31,817(37.68)

**Table 3:** The sociodemographic and institutional characteristics of the sampling inpatients’ recognition in China

	Recognition(%)				
	Definitely won't	Won't	Average	Will	Definitely will
**Sex**					
Male	100(0.12)	279(0.33)	2,659(3.15)	22,173(26.26)	15,928(18.86)
Female	113(0.13)	277(0.33)	2,610(3.09)	23,533(27.87)	16,770(19.86)
**Age**					
Under 18	25(0.03)	37(0.04)	429(0.51)	3,274(3.88)	2,586(3.06)
19–39	55(0.07)	136(0.16)	1,258(1.49)	8,661(10.26)	6,818(8.07)
40–59	65(0.08)	197(0.23)	1,782(2.11)	15,669(18.56)	10,737(12.72)
Above 60	68(0.09)	186(0.22)	1800(2.13)	18,102(21.44)	12,557(14.87)
**Residence**					
Local city	185(0.22)	455(0.54)	4,457(5.28)	40,427(47.88)	27,348(32.39)
Other cities in the province	7(0.01)	25(0.03)	290(0.34)	2,583(3.06)	2,490(2.95)
Other provinces	18(0.02)	76(0.09)	501(0.59)	2,655(3.14)	2,820(3.34)
Hong Kong, Macao Taiwan	0(0.00)	0(0.00)	14(0.02)	26(0.03)	23(0.03)
Overseas	3(0.00)	0(0.00)	7(0.01)	15(0.02)	17(0.02)
**Insurance**					
Urban employee medical insurance	153(0.18)	356(0.42)	2,893(3.43)	21,201(25.11)	14,160(16.77)
Medical insurance for urban and rural residents	46(0.05)	136(0.16)	1,744(2.07)	20,747(24.57)	14,798(17.52)
Public funded health care	1(0.00)	4(0.00)	45(0.05)	188(0.22)	181(0.21)
Commercial insurance	0(0.00)	3(0.00)	22(0.03)	156(0.18)	141(0.17)
Out of pocket	8(0.01)	19(0.02)	273(0.32)	1,557(1.84)	1,117(1.32)
Others	5(0.01)	38(0.05)	292(0.35)	1,857(2.20)	2,301(2.72)
**Occupation**					
Students	134(0.16)	283(0.34)	2,127(2.52)	11,175(13.23)	4,587(5.43)
Company employees	8(0.01)	22(0.03)	293(0.35)	2,996(3.55)	3,110(3.68)
Corporate executives	2(0.00)	3(0.00)	38(0.05)	337(0.40)	340(0.40)
Workers	3(0.00)	27(0.03)	293(0.35)	2,694(3.19)	2,634(3.12)
Farmers	25(0.03)	68(0.08)	792(0.94)	9,595(11.36)	6,453(7.64)
Civil servants	1(0.00)	4(0.00)	56(0.07)	375(0.44)	628(0.74)
Military personnel	0(0.00)	4(0.00)	17(0.02)	126(0.15)	360(0.43)
Individual operation	1(0.00)	10(0.01)	73(0.09)	734(0.87)	802(0.95)
Unemployed	8(0.01)	18(0.02)	294(0.35)	2,682(3.18)	1,673(1.98)
Retired, self-employed, others	31(0.04)	117(0.14)	1,286(1.52)	14,992(17.75)	12,111(14.34)
**Income**					
Under 30,000 yuan	173(0.20)	408(0.48)	3700(4.38)	28,020(33.18)	16,648(19.72)
30,000 −100,000 yuan	32(0.04)	122(0.14)	1,187(1.41)	13,989(16.57)	12,036(14.25)
100,000–200,000 yuan	4(0.00)	20(0.02)	301(0.36)	3,074(3.64)	3,007(3.56)
200,000–500,000 yuan	1(0.00)	6(0.01)	61(0.07)	468(0.07)	776(0.92)
500,000 yuan and above	3(0.00)	0(0.00)	20(0.02)	155(0.18)	231(0.27)
**Reason**					
The hospital is well-known	12(0.01)	41(0.05)	540(0.64)	7,330(8.68)	8,003(9.48)
The high technology, and advanced equipment	18(0.02)	97(0.11)	905(1.07)	10,912(12.92)	8,928(10.57)
Good attitude	17(0.02)	58(0.07)	438(0.52)	5,155(6.10)	5,590(6.62)
Good environment	14(0.02)	43(0.05)	383(0.45)	2,527(2.99)	1,312(1.55)
Nearby	53(0.06)	126(0.15)	1,487(1.76)	12,841(15.21)	6,202(7.34)
Reasonable fees	8(0.01)	49(0.06)	249(0.29)	1,475(1.75)	526(0.62)
Introduced by other people, there are acquaintances in the hospital, others	91(0.11)	142(0.17)	1,267(1.50)	5,466(6.47)	2,137(2.53)
**Referral**					
Referral from higher level hospitals	131(0.16)	278(0.33)	2,014(2.39)	10,057(11.91)	3,574(4.23)
Referral from hospitals of the same level	5(0.01)	9(0.01)	113(0.13)	769(0.91)	883(1.05)
Referral from lower level hospitals	3(0.00)	15(0.02)	142(0.17)	1,088(1.29)	1,182(1.40)
Referral from community clinics	1(0.00)	4(0.00)	34(0.04)	221(0.26)	174(0.21)
Directly come to the hospital	73(0.09)	250(0.30)	2,965(3.51)	33,571(39.76)	26,885(31.84)
**Level**					
Tertiary hospital	8(0.01)	17(0.02)	221(0.26)	960(1.14)	198(0.23)
Secondary hospital	115(0.14)	220(0.26)	2,215(2.62)	18,423(21.82)	8,632(10.22)
Community health centers	90(0.11)	319(0.38)	2,833(3.35)	26,323(31.17)	23,868(28.27)
**Type**					
Gynecology hospital	8(0.01)	14(0.02)	204(0.24)	1,583(1.87)	945(1.12)
Traditional Chinese medicine hospital	5(0.01)	7(0.01)	167(0.20)	1,529(1.81)	787(0.93)
General hospitals	183(0.22)	527(0.62)	4,761(5.64)	41,340(48.96)	30,181(35.74)
Tumor hospital	2(0.00)	5(0.01)	66(0.08)	1,035(1.23)	667(0.79)
Other	15(0.02)	3(0.00)	71(0.08)	219(0.26)	118(0.14)
**Ownership**					
Private	18(0.02)	39(0.05)	431(0.51)	2,807(3.32)	2,136(2.53)
Public	195(0.23)	517(0.61)	4,838(5.73)	42,899(50.80)	30,562(36.19)

**Table 4:** The sociodemographic and institutional characteristics of the sampling inpatients’ recommendation in China

	Recommendation(%)				
	Definitely won't	Won't	Average	Will	Definitely will
**Sex**					
Male	60(0.07)	390(0.46)	2,820(3.34)	22,424(26.56)	15,444(18.29)
Female	62(0.07)	341(0.40)	2,935(3.48)	23,814(28.20)	16,150(19.13)
**Age**					
Under 18	17(0.02)	59(0.07)	462(0.55)	3,309(3.92)	2,504(3.92)
19–39	29(0.03)	174(0.21)	1,341(1.59)	8,694(10.30)	6,690(7.92)
40–59	30(0.04)	239(0.28)	1,898(2.25)	15,966(18.91)	10,317(12.22)
Above 60	46(0.06)	259(0.31)	2,054(2.43)	18,269(21.64)	12,083(14.31)
**Residence**					
Local city	99(0.12)	608(0.72)	5,022(5.95)	40,765(48.28)	26,376(31.24)
Other cities in the province	4(0.00)	33(0.04)	286(0.34)	2,646(3.13)	2,426(2.87)
Other provinces	14(0.02)	89(0.11)	429(0.51)	2,782(3.29)	2,756(3.26)
Hong Kong, Macao Taiwan	2(0.00)	0(0.00)	15(0.02)	28(0.03)	18(0.02)
Overseas	3(0.00)	1(0.00)	3(0.00)	17(0.02)	18(0.02)
**Insurance**					
Urban employee medical insurance	75(0.09)	446(0.53)	3,276(3.88)	21,275(25.20)	13,689(16.21)
Medical insurance for urban and rural residents	31(0.04)	204(0.24)	1,865(2.21)	21,066(24.95)	14,305(16.94)
Public funded health care	0(0.00)	4(0.00)	51(0.06)	185(0.22)	179(0.21)
Commercial insurance	1(0.00)	7(0.01)	29(0.03)	153(0.18)	132(0.16)
Out of pocket	11(0.01)	28(0.03)	263(0.31)	1,619(1.92)	1,053(1.25)
Others	4(0.00)	42(0.05)	271(0.32)	1,940(2.30)	2,236(2.65)
**Occupation**					
Students	66(0.08)	321(0.38)	2,274(2.69)	11,184(13.24)	4,461(5.28)
Company employees	3(0.00)	38(0.05)	413(0.49)	2,943(3.49)	3,031(3.59)
Corporate executives	2(0.00)	4(0.00)	42(0.05)	343(0.41)	329(0.39)
Workers	3(0.00)	32(0.04)	305(0.36)	2,764(3.27)	2,546(3.02)
Farmers	19(0.02)	93(0.11)	839(0.99)	9,781(11.58)	6,201(7.34)
Civil servants	0(0.00)	5(0.01)	60(0.07)	394(0.47)	605(0.72)
Military personnel	0(0.00)	3(0.00)	22()0.03	134(0.16)	348(0.41)
Individual operation	1(0.00)	12(0.01)	79(0.09)	763(0.90)	765(0.91)
Unemployed	7(0.01)	36(0.04)	289(0.34)	2,737(3.24)	1,606(1.90)
Retired, self-employed, others	21(0.02)	187(0.22)	1,432(1.70)	15,195(18.00)	11,702(13.86)
**Income**					
Under 30,000 yuan	93(0.11)	512(0.61)	3,947(4.67)	28,383(33.61)	16,013(18.96)
30,000 −100,000 yuan	23(0.03)	179(0.21)	1,414(1.67)	14,120(16.72)	11,629(13.77)
100,000–200,000 yuan	5(0.01)	27(0.03)	308(0.36)	3,110(3.68)	2,956(3.50)
200,000–500,000 yuan	1(0.00)	8(0.01)	68(0.08)	459(0.54)	776(0.92)
500,000 yuan and above	0(0.00)	5(0.01)	18(0.02)	166(0.20)	220(0.26)
**Reason**					
The hospital is well-known	6(0.01)	51(0.06)	592(0.70)	7,508(8.89)	7,769(9.20)
The high technology, and advanced equipment	9(0.01)	92(0.11)	892(1.06)	11,151(13.21)	8,715(10.32)
Good attitude	6(0.01)	64(0.08)	456(0.54)	5,376(6.37)	5,356(6.34)
Good environment	8(0.01)	51(0.06)	411(0.49)	2,565(3.04)	1,243(1.47)
Nearby	31(0.04)	219(0.26)	1,814(2.15)	12,752(15.10)	5,893(6.98)
Reasonable fees	0(0.00)	47(0.06)	323(0.38)	1,438(1.70)	499(0.59)
Introduced by other people, there are acquaintances in the hospital, others	62(0.07)	207(0.25)	1,267(1.50)	5,448(6.45)	2,119(2.51)
**Referral**					
Referral from higher level hospitals	66(0.08)	317(0.38)	2,150(2.55)	10,033(11.88)	3,488(4.13)
Referral from hospitals of the same level	2(0.00)	15(0.02)	101(0.12)	808(0.96)	853(1.01)
Referral from lower level hospitals	4(0.00)	24(0.03)	126(0.15)	1,117(1.32)	1,160(1.37)
Referral from community clinics	0(0.00)	4(0.00)	33(0.04)	229(0.27)	168(0.20)
Directly come to the hospital	50(0.06)	371(0.44)	3,345(3.96)	34,051(40.33)	25,925(30.70)
**Level**					
Tertiary hospital	2(0.00)	14(0.02)	229(0.27)	965(1.14)	194(0.23)
Secondary hospital	70(0.08)	327(0.39)	2,524(2.99)	18,474(21.88)	8,210(9.72)
Community health centers	50(0.06)	390(0.46)	3,002(3.56)	26,799(31.74)	23,190(27.46)
**Type**					
Gynecology hospital	4(0.00)	25(0.03)	215(0.25)	1,583(1.87)	927(1.10)
Traditional Chinese medicine hospital	3(0.00)	19(0.02)	186(0.22)	1,549(1.83)	738(0.87)
General hospitals	103(0.12)	664(0.79)	5,245(6.21)	41,805(49.51)	29,173(34.55)
Tumor hospital	1(0.00)	7(0.01)	62(0.07)	1,056(1.25)	649(0.77)
Other	11(0.01)	16(0.02)	47(0.06)	245(0.29)	107(0.13)
**Ownership**					
Private	9(0.01)	51(0.06)	465(0.55)	2,830(3.35)	2,076(2.46)
Public	113(0.13)	680(0.81)	5,290(6.26)	43,408(51.41)	29,518(34.96)

### The people-centered care

Table 5 shows the people-centered care experienced by the sampled patients. When examining the first attribute of people-centered care, 58.53% of the participants experienced a lower level of continuity of health services, and 41.47% of them experienced a higher level of continuity. Among the total sampled participants with lower levels of continuity of care, 48.03% were satisfied with the full-length process during the hospitalization, 46.69% were willing to come to the same hospital when they needed subsequent health services, and 46.51% were willing to recommend the hospital to their friends and relatives. When examining the participants who experienced a higher level of continuity of care, 11.65% were satisfied with the entire process when searching for health services, 7.44% were willing to come to the same hospital again and 8.25% were willing to recommend the hospital.

**Table 5:** Patient perceived experience across different types of people-centered care among the sampling inpatients in China

	Satisfaction(%)					Recognition(%)					Recommendation(%)				
	Very dissatisfied	Dissatisfied	Average	Satisfied	Very satisfied	Definitely won't	Won't	Average	Will	Definitely will	Definitely won't	Won't	Average	Will	Definitely will
**Continuity**															
Lower level	70(0.08)	284(0.34)	3,968(4.70)	35,886(42.50)	4,115(4.87)	195(0.23)	517(0.61)	4,842(5.73)	39,427(46.69)	4,441(5.26)	117(0.14)	692(0.82)	5,318(6.30)	39,273(46.51)	4,022(4.76)
High level	16(0.02)	16(0.02)	338(0.40)	9,833(11.65)	29,912(35.42)	18(0.02)	39(0.05)	427(0.51)	6,279(7.44)	28,257(33.46)	5(0.01)	39(0.05)	437(0.52)	6,965(8.25)	27,572(32.65)
**Information sharing**															
Lower level	70(0.08)	284(0.34)	3,968 (4.70)	35,886(42.50)	4,115 (4.87)	178(0.21)	481(0.57)	4,671(5.53)	34,879(41.31)	4,113(4.87)	109(0.13)	660(0.78)	5,062(5.99)	34,691(41.08)	3,800(4.50)
High level	16(0.02)	16(0.02)	338 (0.40)	9,833 (11.65)	29,912(35.42)	35(0.04)	75(0.09)	598(0.71)	10,827(12.82)	28,585(33.85)	13(0.02)	71(0.08)	693(0.82)	11,547(13.67)	27,794(32.92)
**Enhanced access**															
Lower level	72(0.09)	286 (0.34)	4,041 (4.79)	36,355(43.06)	4,667(5.53)	181(0.21)	499(0.59)	4,691(5.56)	35,383(41.90)	4,667(5.53)	108(0.13)	674(0.80)	5,094(6.03)	35,188(41.67)	4,358(5.16)
High level	14(0.02)	14(0.02)	265 (0.31)	9,364 (11.09)	29,360(34.77)	32(0.04)	57(0.07)	578(0.68)	10,323(12.22)	28,031(33.20)	14(0.02)	57(0.07)	661(0.78)	11,050(13.09)	27,236(32.25)
**Effectiveness**															
Lower level	70(0.08)	273 (0.32)	3,980 (4.71)	35,321(41.83)	3,202 (3.79)	172(0.20)	480(0.57)	4,600(5.45)	34,236(40.54)	3,358(3.98)	107(0.13)	657(0.78)	5,008(5.93)	34,065(40.34)	3,010(3.56)
High level	16 (0.02)	27 (0.03)	326 (0.39)	10,398(12.31)	30,825(36.51)	41(0.05)	76(0.09)	669(0.79)	11,470(13.58)	29,340(34.75)	15(0.02)	74(0.09)	747(0.88)	12,173(14.42)	28,584(33.85)
**Respect**															
Lower level	75 (0.09)	286 (0.34)	4,072 (4.82)	38,060(45.07)	3,437 (4.07)	186(0.22)	498(0.59)	4,754(5.63)	36,861(43.65)	3,631(4.30)	110(0.13)	669(0.79)	5,205(6.16)	36,604(43.35)	3,342(3.96)
High level	11 (0.01)	14 (0.02)	234 (0.28)	7,659 (9.07)	30,590(36.23)	27(0.03)	58(0.07)	515(0.61)	8,845(10.47)	29,067(34.42)	12(0.01)	62(0.07)	550(0.65)	9,634(11.41)	28,252(33.46)

When examining information sharing, 52.49% of the participants’ evaluation of the level of information sharing is considered to be lower class, whereas 47.51% of them experienced a higher level of information sharing. Of all the participants, 42.50% were satisfied with the service-seeking process, 41.31% agreed that they would come to the same hospital and 41.08% were willing to recommend the hospital that considered their level of information sharing during the health services-seeking process to be low. In terms of the participants experiencing a higher level of information sharing, 11.65% of the total sampled inpatients were satisfied with the entire hospitalization process, 12.82% were willing to come to the hospital if they need subsequent health services, and 13.67% were willing to recommend the hospital.

In terms of enhanced access, 53.79% of the participants of the survey evaluated the access to health services at a low level whereas 46.21% of them thought the access to health services was considered to be of a higher level. Among the participants experiencing lower levels of access to health services, 43.06% of them were satisfied with the entire process of the hospitalization, 41.90% were willing to come to the same hospital for subsequent health services, and 41.67% were willing to recommend the hospital. In the case of the inpatients with higher levels of access to health services, 11.09% of the entire sample of inpatients were satisfied with the full-length process when hospitalized, 12.22% agreed that they would come to the same hospital and 13.09% were willing to make the recommendation of the hospital to their friends and relatives.

When taking the fourth attribute into consideration, the effectiveness of the health services, 50.74% of the total sampled inpatients rated it at a lower level and 49.26% rated it at a higher level. Of the inpatients who experienced lower levels of effectiveness of the health services, 41.83% of the total participants were satisfied with the overall process of the hospitalization, 40.54% of them would certainly come to the same hospital when needed and 40.34% would decidedly recommend the hospital. As far as the inpatients who experienced a higher level of effectiveness of the health services, 12.31% of the entirety of the sampled inpatients were satisfied with the full-length process, 13.58% stated they will come to the same hospital, and 14.42% will recommend the hospital.

In terms of the respect that the participants experienced, 54.39% of the participants thought they experienced a lower level of respect and 45.61% of them experienced a higher level of respect. In the case of the participants who experienced a lower level of respect, 45.07% of the total sampled inpatients were satisfied with the hospitalization experience, 43.65% of them would come to the hospital again and 43.35% would recommend the hospital. As for the participants with higher experience of respect, 9.07% of them were satisfied with the process when hospitalized, 10.47% of the participants would come to the hospital again and 11.41% of them believed that they would recommend the hospital to their friends and relatives.

### Association between people-centered care and inpatients’ perceived experience

Table 6 shows the results from the proportional odds models that show the relationship between each PCC attribute individually and the patients’ perceived experience. Higher levels of continuity, information sharing, enhanced access, effectiveness, and respect were positively associated with the enhancement of the patients’ experience including satisfaction, recognition, and recommendation. The higher level of PCC particularly has a larger effect on the improvement of the patients’ perceived experience from “satisfied” to “very satisfied” or from “will” to “definitely will.” The results also presented that continuity has a significant effect on the improvement of the patients’ perceived experience when compared to other attributes of PCC.

**Table 6:** Association between each individual PCC domain and inpatients’ perceived experience among the sampling inpatients in China

	Satisfaction				Recognition				Recommendation			
	(1vs2,3,4,5)	(1,2vs2,3,4,5)	(1,2,3vs4,5)	(1,2,3,4vs5)	(1vs2,3,4,5)	(1,2vs2,3,4,5)	(1,2,3vs4,5)	(1,2,3,4vs5)	(1vs2,3,4,5)	(1,2vs2,3,4,5)	(1,2,3vs4,5)	(1,2,3,4vs5)
	OR	OR	OR	OR	OR	OR	OR	OR	OR	OR	OR	OR
**Continuity**	5.93***	13.03***	14.69***	52.58***	4.74***	6.25***	7.25***	38.76***	9.03***	9,47***	8.24***	38.47***
**Information sharing**	2.66***	7.9***	9.57***	26.24***	3.05***	3.77***	5.92***	22.11***	4.88***	5.82***	6.04***	22.16***
**Enhanced access**	2.42***	8.31***	11.69***	24.19***	2.57***	4.43***	6.01***	19.99***	3.88***	6.45***	6.15***	19.73***
**Effectiveness**	2.73***	6.07***	10.61***	32.11***	2.43***	3.90***	5.83***	25.33***	4.67***	5.95***	6.16***	26.35***
**Respect**	4.23***	10.34***	13.22***	45.46***	3.18***	4.77***	6.76***	32.49***	5.58***	6.28***	7.32***	31.84***

Note: *** Indicates significance at the 1% significance level

Table 7 shows the results of the proportional odds models including all the attributes of the PCC and the entirety of the covariates.

**Table 7:** Association between the people-centered care and inpatients’ perceived experience among the sampling inpatients in China

	Satisfaction				Recognition				Recommendation			
	(1vs2,3,4,5)	(1,2vs2,3,4,5)	(1,2,3vs4,5)	(1,2,3,4vs5)	(1vs2,3,4,5)	(1,2vs2,3,4,5)	(1,2,3vs4,5)	(1,2,3,4vs5)	(1vs2,3,4,5)	(1,2vs2,3,4,5)	(1,2,3vs4,5)	(1,2,3,4vs5)
	OR	OR	OR	OR	OR	OR	OR	OR	OR	OR	OR	OR
**Continuity**	3.66**	2.28**	1.88***	6.78***	2.51***	2.31***	1.63***	5.80***	3.41**	2.72	1.90***	5.89***
**Information sharing**	0.62	1.83**	1.54***	1.91***	1.23	1.14	1.56***	1.93***	2.12	1.42**	1.49***	1.93***
**Enhanced access**	0.69	1.96***	2.22***	1.77***	1.08	1.57***	1.58***	1.65***	0.77	1.72***	1.51***	1.60***
**Effectiveness**	1.97	1.14	2.19***	2.62***	0.97	1.30*	1.66***	2.24***	1.45	1.69***	1.71***	2.42***
**Respect**	2.17	2.50***	2.44***	3.80***	1.40	1.54**	1.88***	3.13***	1.50	1.34*	1.92***	2.91***
**Sex**	0.76	0.98	0.99	1.04	0.90	1.00	1.08***	1.06***	0.96	1.19**	1.04	1.04*
**Age**	0.98	1.01	1.11	0.99	1.21***	1.11***	1.10***	1.00	1.15	1.10***	1.07***	0.99
**Residence**	0.73**	0.83**	0.90	1.03	0.97	0.86***	0.86***	1.06***	0.72***	0.82***	0.94***	1.08***
**Insurance**	0.89	0.89***	0.92	1.04***	0.95	0.90***	0.90***	1.03***	0.87*	0.94**	0.95***	1.02*
**Occupation**	1.03	1.01	1.00	1.00	1.03	1.01	1.01	1.00	0.99	0.98*	1.02***	1.00
**Income**	1.01	0.94	1.03	1.09***	0.94	1.05	1.03	1.10***	0.98	1.00	1.00	1.13***
**Reason**	0.73***	0.87***	0.92	0.97***	0.77***	0.85***	0.87***	0.93***	0.67***	0.79***	0.85***	0.94***
**Referral**	1.29***	1.21***	1.14	0.94***	1.35***	1.32***	1.17***	0.96***	1.25***	1.22***	1.11***	0.95***
**Level**	1.03	0.86	0.96	1.23***	1.15	0.90	0.99	1.17***	1.15	0.94	1.03	1.22***
**Type**	0.54***	0.69***	0.96	1.02	0.58***	0.86**	0.98	1.04	0.59***	0.83**	1.03	1.00
**Public**	0.29**	0.56**	1.01	1.00	0.65*	0.74**	0.96	0.95	0.82	0.80	0.94	0.93*
**Area**	0.97	1.05	0.99	0.97***	1.05	1.00	0.98**	0.96***	0.92	1.01	1.01*	0.96***

Note:** indicates significance at the 5% significance level, and *indicates significance at the 10% significance level

When examining the inpatients’ overall satisfaction with the hospitalization, continuity was significantly linked with a higher overall satisfaction with the hospitalization. When comparing the inpatients who experienced a lower level of continuity, the inpatients who experienced a higher level of continuity were 3.66 times more likely to improve their level of satisfaction from “very unsatisfied” to “unsatisfied,” 2.28 times more likely to improve the satisfaction from “unsatisfied” to “average,” 1.88 times more likely to improve the satisfaction from “average” to “satisfied,” and 1.88 times more likely to improve the satisfaction from “satisfied” to “very satisfied.” Compared with the inpatients who experienced a lower level of information sharing, the inpatients who experienced a higher level of information sharing were 1.83 times more likely to improve their level of satisfaction from “unsatisfied” to “average,” 1.54 times more likely to improve their level of satisfaction from “average” to “satisfied,” and 1.91 times more likely to improve their level of satisfaction from “satisfied” to “very satisfied.” The effect of information sharing on the amelioration of overall satisfaction from “very unsatisfied” to “unsatisfied” is not significant. Compared to the inpatients who experienced a lower level of enhanced access to health services, the inpatients who experienced a higher level of enhanced access were 1.96 times more likely to improve their level of satisfaction from “unsatisfied” to “average,” 2.22 times more likely to improve their level of satisfaction from “average” to “satisfied,” and 1.77 times more likely to improve their level of satisfaction from “satisfied” to “very satisfied.” The effect of enhanced access on the improvement of overall satisfaction from “very unsatisfied” to “unsatisfied” is not significant. When considering the case of effectiveness, compared to the inpatients who experienced a lower level of effectiveness of health services, the inpatients who experienced a higher level of effectiveness were 2.22 times more likely to improve their level of satisfaction from “average” to “satisfied,” and 1.77 times more likely to improve their level of satisfaction from “satisfied” to “very satisfied.” The effect of the effectiveness of health services on the improvement of overall satisfaction is not significant from “very unsatisfied” to “unsatisfied” and “unsatisfied” to “average.” When examining respect in healthcare settings, compared to the inpatients who experienced a lower level of respect, the inpatients who experienced a higher level of respect were 2.50 times more likely to improve their level of satisfaction from “unsatisfied” to “average,” 2.44 times more likely to improve their level of satisfaction from “average” to “satisfied,” and 3.80 times more likely to improve their level of satisfaction from “satisfied” to “very satisfied.” The effect of respect on the improvement of overall satisfaction from “very unsatisfied” to “unsatisfied” is not significant.

In consideration of the inpatients’ recognition, continuity was significantly associated with the higher overall satisfaction of the hospitalization. In comparison with the inpatients who experienced a lower level of continuity, the inpatients who experienced a higher level of continuity were 2.51 times more likely to improve the recognition, from “definitely won't come again” to “won't come again,” 2.31 times more likely to improve the recognition, from “won't come again” to “average,” 1.63 times more likely to improve the recognition, from “average” to “will come again” and 5.80 times more likely to improve the recognition from “will come again” to “definitely will come again.” Compared with the inpatients who experienced a lower level of information sharing, the inpatients who experienced a higher level of information sharing were 1.56 times more likely to improve the recognition from “average” to “will come again,” and 1.93 times more likely to improve the recognition from “will come again” to “definitely will come again.” The effects of enhanced access on the improvement of identification from “definitely won't come again” to “won't come again” lack significance. The inpatients who experienced a higher level of information sharing were 1.57 times more likely to improve the recognition from “won't come again” to “average,” 1.58 times more likely to improve the recognition from “average” to “will come again,” and 1.65 times more likely to improve the recognition from “will come again” to “definitely will come again.” In the case of effectiveness, compared to the inpatients who experienced a lower level of effectiveness of health services, the inpatients who experienced a higher level of effectiveness are 1.30 times more likely to improve the recognition from “won't come again” to “average,” 1.66 times more likely to improve the recognition from “average” to “will come again,” 2.24 times more likely to improve the recognition from “will come again” to “definitely will come again.” The effect of the effectiveness of health services on the improvement of overall recognition is not significant from “definitely won't come again” to “won't come again.” When examining respect in healthcare settings, compared to the inpatients who experienced a lower level of respect, the inpatients who experienced a higher level of respect were 1.54 times more likely to improve the recognition from “won't come again” to “average,” 1.88 times more likely to improve the recognition from “average” to “will come again,” and 3.13 times more likely to improve the recognition from “will come again” to “definitely will come again.” The effect of respect on the improvement of recognition from “definitely won't come again” to “won't come again” lacks significance.

As far as the inpatients’ recommendation measures, compared with the inpatients who experienced a lower level of continuity, the inpatients who experienced a higher level of continuity were 3.41 times more likely to improve the recommendation from “definitely won't recommend” to “won't recommend,” 1.90 times more likely to improve the recommendation from “average” to “will recommend,” and 5.89 times more likely to improve the recommendation from “will recommend” to “definitely will recommend.” Compared with the inpatients who experienced a lower level of information sharing, the inpatients who experienced a higher level of information sharing were 1.42 times more likely to improve the recommendation from “won't recommend” to “average,” 1.49 times more likely to improve the recommendation from “average” to “will recommend” and 1.93 times more likely to improve the recommendation from “will recommend” to “definitely will recommend.” The effects of information sharing on the improvement of recommendations from “definitely won't recommend” to “won't recommend” lack significance. Compared to the inpatients who experienced a lower level of enhanced access to health services, the inpatients who experienced a higher level of enhanced access were 1.72 times more likely to improve the recommendation from “won't recommend” to “average,” 1.51 times more likely to improve the recommendation from “average” to “will recommend” and 1.60 times more likely to improve the recommendation from “will recommend” to “definitely will recommend.” The effects of enhanced access on the likelihood of patient recommendation from “definitely won't recommend” to “won't recommend” lacks significance. In the case of effectiveness, compared to the inpatients who experienced a lower level of effectiveness of health services, the inpatients who experienced a higher level of effectiveness were observed to have a 6.9% higher likelihood of enhancing their survey answer from “won't recommend” to “average,” and inpatients with a higher level of effectiveness were 1.51 times more likely to improve the recommendation from “average” to “will recommend,” and 1.60 times more likely to improve the recommendation from “will recommend” to “definitely will recommend.” The effects of enhanced access on the improvement of patient recommendations from “definitely won't recommend” to “won't recommend” lacks significance. In the case of respect, compared to the inpatients who experienced a lower level of respect, the inpatients who experienced a higher level of respect were 1.34 times more likely to improve the recommendation from “won't recommend” to “average,” 1.92 times more likely to improve the recommendations from “average” to “will recommend” and 2.91 times more likely to improve the recommendation from “will recommend” to “definitely will recommend.” The effects of enhanced access on the improvement of recommendations from “definitely won't recommend” to “won't recommend” lacks significance.

## Discussion

Improving the patient experience is a crucial measure to promote a patient’s level of health, and improve the efficiency, quality, level of medical services and health equity [38]. With the progress of China's new healthcare reform, PCC has gradually become the center of China's health policies and reforms. A significant number of healthcare reform measures aim to promote PCC, empower patients, and fortify the role of patients in medical service production. Studying the impact of PCC on patient experience is of tremendous significance for further promoting healthcare reform, and improving the quality, fairness, and accessibility of medical services.

PCC is an intricate and complex concept, and various countries have developed assorted conceptual and measurement models for PCC based on their own characteristics [39, 40]. Based on the existing research results, China's reform practices, and the characteristics of the data, the study used five indicators, continuity, information sharing, enhanced access, effectiveness, and respect to measure PCC. Our research discovered that the relationship between various indicators and the patients’ perceived experience exists strong relevance, which implies that the inherited effect of PCC on the patients’ perceived experience is compounded and unable to be understood in some kind of fixed pattern, which means that the PCC is a complex and comprehensive concept and the effect of PCC should be studied with the understand of different attributes of PCC and corresponding context of different countries and areas [41–43].

As can be clearly observed through this study, it was discovered that generally speaking, the PCC, including care continuity, information sharing, enhanced access, effectiveness and respect has a positive effect on the patient perceived experience including the inpatients’ satisfaction, recognition and recommendation, and the magnitude of this effect varies with the degree of improvement in the patient perceived experience. When taking into consideration all the indicators, it was discovered that continuity has a greater impact on satisfaction, recognition, and recommendation on the patient perceived experience compared to other indicators, indicating that service continuity has a significant impact on the patient's experience. This discovery provides evidence-based support for further promoting reforms such as graded diagnosis and treatment and integrated care in China. Simultaneously, information sharing, enhanced access, effectiveness, and respect have a positive effect on improving the patient experience as well. This leads credibility to the idea that subsequent policies and practices should focus on protecting the patient's right to know, enhancing respect and understanding for patients, and aiming for the continuous improvement of the quality of medical services.

PCC places an emphasis on the joint role of patients, their families, and communities in decision-making and service provision [44, 45]. Stated in different terms, patients and residents should become the "first responsible persons" for their individual health and engage in a more decisive role in health production [45]. This requires sufficient communication, understanding, and trust between patients and doctors [46–48]. Nevertheless, China is currently continuously promoting the general practitioners signing system, strengthening the connection between residents and GPs, regulating patient behavior, and enhancing patient participation in decision-making through this system.

The sample of this study encompasses tertiary hospitals, secondary hospitals, and community health service centers in 31 provinces in eastern, central, and western China. The types of hospitals also include gynecology hospitals, traditional Chinese medicine hospitals, general hospitals, tumor hospitals, and others, and the data has a high degree of timeliness and is considered to have widespread coverage. This nationwide study indicates that PCC, including the attributes of continuity, information sharing, enhanced access, effectiveness, and respect, has a positive effect on patient perceived experience, and this effect is more pronounced inpatients’ levels of satisfaction that range from “satisfied” to “very satisfied” or from “will” to “definitely will.”, which aligns with existing literature [49–51] For healthcare settings such as hospitals, this means a focus on continuous improvement of PCC should be a priority in order to further enhance patient experience.

Among the 5 attributes of PCC, it was discovered that continuity has a more significant impact on the improvement of the patient experience, which leads credibility to the idea that promoting the connection between medical services is essential, and this includes the connection between various levels of medical institutions, as well as the efficiency of patient visits and hospitalizations after arrival, all of which play a key role in the patient experience. Simultaneously, promoting information sharing between doctors and patients, promoting a patient’s right to know and enabling the patient to participate in their individual health production process, and fully respecting patients have taken on an essential role in improving patient satisfaction as well. Simultaneously, the quality, access, and effectiveness of services also maintain a pivotal role, which gives evidence to the importance of continuously improving hospital service capabilities.

Nonetheless, China also faces a number of problems on the path toward improving PCC, health equity, and patient experience. Due to the immaturity of China’s current referral system, patients can choose to visit any institution they prefer, which leads to a concentration of patients in tertiary hospitals, ultimately resulting in overcrowding in these hospitals. Due to the excessive number of patients, the time for communication between healthcare professionals, such as doctors, and patients is reduced, and the respect for patients may also be compromised. This could have a negative impact on patients' medical experience. Consistent with the existing research findings, this study indicate that PCC is crucial for patient satisfaction, recognition, and recommendation. Therefore, improving PCC has become an important part of hospital management and health reform in China. This further emphasizes that the understanding and recognition of PCC must be placed within the broader context of health reform [52].

The current focus of China's health reform is to establish a county healthcare system, which strengthens the connections between medical institutions at different levels and introduces economic incentives to encourage patients to follow the recommendations of general practitioners and seek care in the order of primary, secondary, and tertiary healthcare institutions. This reform helps to reduce overcrowding in tertiary and secondary hospitals, providing doctors and other healthcare professionals with more time and space to communicate with patients. It also enhances the involvement of patients and their families in medical decision-making, thereby improving PCC and ultimately having a positive impact on patients' health outcomes and hospital experience. At the same time, the current reform of public hospitals in China also provides the foundation and environment for enhancing Patient—Centered Care (PCC). The reform of public hospitals requires the provision of high—quality and efficient services to patients. This can be achieved through means such as conducting patient satisfaction surveys and establishing electronic health records for patients, so as to improve the level of patients' participation in decision—making and information sharing during the service delivery process.

This study presented the hypothesis that PCC has a significantly positive effect on patients’ perceived experience utilizing national data including 84,438 inpatients from 351 health institutions, which provided the foundation and empirical evidence for a future reform aimed at the promotion of PCC in China and around the world. Even so, this study has limitations in that one year of data is unable to support the causal inference of PCC and patients perceived experience, which necessitates and calls for future studies to explore the causal relationship between the two variables.

This study has some limitations. The measurement of PCC in this study is based on China's current reforms and practices. Given the policy content of relevant reforms in China and the available data, the study did not include the dimension of patient involvement in decision-making in the PCC measurement framework. The PCC framework used in this study is an unvalidated indicator based on the Chinese government health policy,and the measurement of PCC in this study may not be applicable to the context of all countries.

## Conclusion

The findings of this nationwide cross-sectional study suggest that people-centered care is positively associated with patients’ perceived experience including satisfaction, recognition, and recommendation. Further healthcare reforms and healthcare practices should focus on the promotion of continuity of care, information sharing between medical staff and patients, access and effectiveness of care, and an increase in the level of respect shown to patients.

## Data Availability

No datasets were generated or analysed during the current study.
